# Growth Cessation and Dormancy Induction in Micropropagated Plantlets of *Rheum rhaponticum* ‘Raspberry’ Influenced by Photoperiod and Temperature

**DOI:** 10.3390/ijms24010607

**Published:** 2022-12-29

**Authors:** Agnieszka Wojtania, Monika Markiewicz, Piotr Waligórski

**Affiliations:** 1 Department of Applied Biology, The National Institute of Horticultural Research, Konstytucji 3 Maja 1/3 Street, 96-100 Skierniewice, Poland; 2 Department of Biotechnology, The Franciszek Górski Institute of Plant Physiology, Polish Academy of Sciences, Niezapominajek 21 Street, 30-239 Krakow, Poland

**Keywords:** ABA, antioxidant enzymes, heat stress, molecular analysis, rhubarb, starch, stress response

## Abstract

Dormancy development in micropropagated plantlets at the acclimatization stage and early growth ex vitro is undesirable as it lowers their survival rate and restricts the efficient year-round production of planting material. Thus far, little is known about the factors and mechanisms involved in the dormancy development of micropropagated herbaceous perennials, including rhubarb. This study determined physiological and molecular changes in the *Rheum rhaponticum* (culinary rhubarb) ‘Raspberry’ planting material in response to photoperiod and temperature. We found that the rhubarb plantlets that were grown under a 16-h photoperiod (LD) and a temperature within the normal growth range (17–23 °C) showed active growth of leaves and rhizomes and did not develop dormancy. Rapid growth cessation and dormancy development were observed in response to a 10-h photoperiod (SD) or elevated temperature under LD. These morphological changes were accompanied by enhanced abscisic acid (ABA) and starch levels and also the upregulation of various genes involved in carbohydrate synthesis and transport (*SUS3, AMY3*, *BMY3, BGLU17*) and ABA synthesis and signaling (*ZEP* and *ABF2*). We also found enhanced expression levels of heat shock transcription factors (*HSFA2* and *HSFA6B*), heat shock proteins (*HSP22*, *HSP70.1*, *HSP90.2* and *HSP101*) and antioxidant enzymes (*PRX12*, *APX2* and *GPX*). This may suggest that dormancy induction in micropropagated rhubarb plantlets is a stress response to light deficiency and high temperatures and is endogenously coordinated by the ABA, carbohydrate and ROS pathways.

## 1. Introduction

Culinary (garden) rhubarb (*Rheum rhaponticum,* syn. *R. rhabarbarum; Polygonaceae*) is a perennial vegetable grown for its long fleshy petioles. Due to their high content of phenolics (stilbenes, anthocyanins and flavonols), fruit acids and vitamins C and K, they are a valued raw material for direct consumption and the food and pharmaceutical industry [1,2,3]. Consumers and the food industry prefer red stalks for their taste and health-promoting qualities. The red-stalked cultivars and valuable selections should be propagated vegetatively to maintain desirable traits. Conventional propagation by crown division gives a low yield of mother plants (2–6 propagules per plant each year) and the risk of virus transfer. Tissue cultures from virus-indexed rhubarb plants are a valuable alternative for the mass production of virus-free planting material. In vitro propagation methods of culinary rhubarb have been demonstrated for different cultivars and selections, including ‘Victoria’ [4], ‘Karpow Lipskiego’ [5], ‘Big Red’, ‘Cherry Red’ [6] and ‘Malinowy’ (‘Raspberry’) [7]. Thus far, little is known about the ex vitro growth of micropropagated rhubarb plantlets. This stage is crucial due to the intensive growth of both above- and below-ground organs (rhizomes). The plantlets with well-developed rhizomes and secondary root systems show better growth in the plantation, higher resistance to different stresses and, as a result, increased yield quantity and quality. Our previous study has indicated dormancy induction in micropropagated rhubarb plantlets during ex vitro growth in the greenhouse [8].

Bud dormancy is an important survival strategy to overcome periods with unfavorable growth conditions, such as hot dry summers or cold winters [9,10]. Dormancy also occurs frequently at different stages of micropropagation [11,12,13]. Some woody and herbaceous perennials, including walnut, peony and magnolia, tended to develop dormancy during acclimatization and early growth ex vitro [14,15,16]. It results in a poor survival rate of plantlets and consequently restricts tissue culture application for the mass production of planting material [17,18]. Knowledge of the dormancy development in micropropagated plantlets is limited, and its control is essential in horticultural practice.

Temperature and photoperiod are the main environmental signals controlling the seasonal dormancy cycle in perennials [9]. However, the species and cultivars vary significantly in their responses to them. Shortening of the photoperiod (short days; SD) is widely accepted as the primary regulator of growth cessation and winter dormancy induction in many perennial plants of the temperate zone [19,20]. In some plant species, including poplar, temperature modifies sensitivity to daylength signals [21,22]. In contrast, a low temperature only is sufficient for growth cessation and dormancy induction in apples, pears and *Sorbus* [23,24]. Evaluating how these key environmental factors affect the ex vitro growth of rhubarb plantlets is extremely important in developing successful techniques for the efficient year-round production of planting material.

The aim of the study was to determine the influence of photoperiod and temperature on the growth and dormancy development of micropropagated rhubarb ‘Raspberry’ plantlets. Physiological and molecular changes were evaluated to better understand the mechanism of dormancy induction in the rhubarb planting material in response to environmental signals.

## 2. Results

### 2.1. The Effect of Photoperiod and Temperature (Experiment 1)

**Figure 1:**
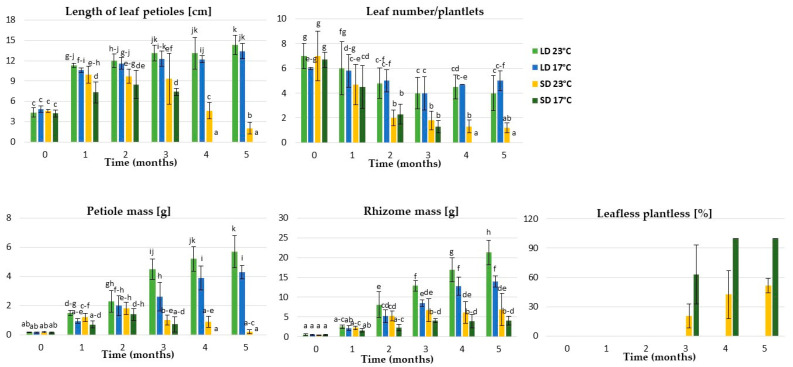
The effect of temperature (17 °C and 23 °C) and photoperiod (LD—long day, SD—short day) on ex vitro growth of micropropagated rhubarb ‘Raspberry’ plantlets after different growth periods (0, 1, 2, 3, 4 and 5 months) in the growth room. According to Duncan’s test (*p* = 0.05), the means marked with the same letter within each growth parameter do not differ significantly. Error bars represent standard deviation.

**Figure 2:**
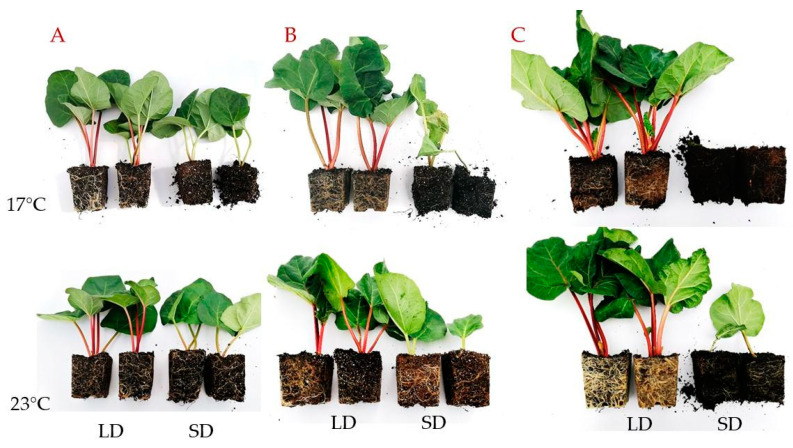
Phenotypic characteristics of the rhubarb ‘Raspberry’ plantlets after different growth periods ((**A**)—2 months, (**B**)—3 months, (**C**)—4 months) in the growth room under different temperatures (17 °C and 23 °C) and photoperiods (16 h and 10 h); LD—long day, SD—short day.

**Figure 3:**
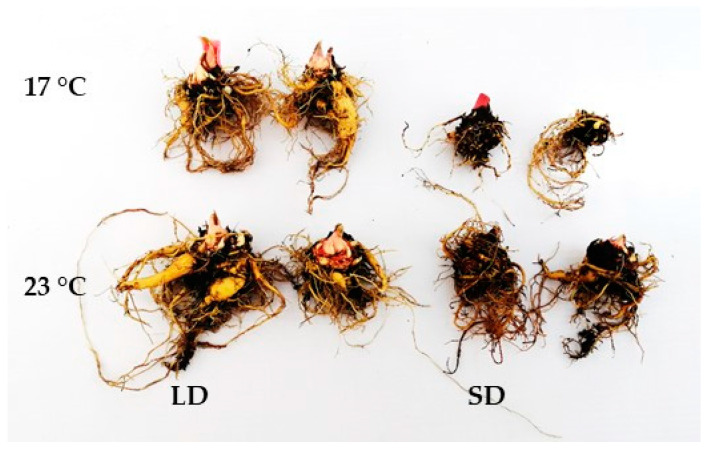
The effect of temperature (17 °C and 23 °C) and photoperiod (LD—long day, SD—short day) on rhizome growth of rhubarb plantlets after three months of growth in the growth room.

The acclimatized plantlets of rhubarb ‘Raspberry’ derived from micropropagation were grown with different photoperiods (16 h and 10 h) and temperatures (17 °C and 23 °C) for five months to determine the ex vitro growth of the planting material. We observed intensive growth of the leaf petioles over the first month of growth in controlled conditions. However, plantlets exposed to a 10-h photoperiod showed rapid growth cessation and dormancy development (Figure 1, Figure 2 and Figure 3). The daylength effect varied depending on temperature (17 °C and 23 °C). At both temperatures, growth cessation in SD started after one month. It was accompanied by a reduced number, length and mass of leaf petioles, as well as rhizome and root system development. A lower temperature increased the rate of SD-induced growth cessation and time (days) to dormancy induction/development. At 17 °C, rapid leaf senescence was observed over the third month, and, at the end of the fourth month, 100% of plantlets were leafless (Figure 2). At 23 °C, 50% of plantlets continued growing after five months in SD, but the growth rate was significantly reduced. In contrast, dormancy did not develop under LD, irrespective of temperature. However, at 23 °C, the fresh mass of leaf petioles and rhizomes was higher than at 17 °C (Figure 1).

#### 2.1.1. Physiological Responses of the Underground Buds during Dormancy Induction

The content of soluble sugars and starch in the vegetative buds of the rhubarb ‘Raspberry’ was determined to characterize the physiological changes that occurred during five-month growth in the growth room under different photoperiods (16 h and 10 h) and temperatures (17 °C and 23 °C).

**Figure 4:**
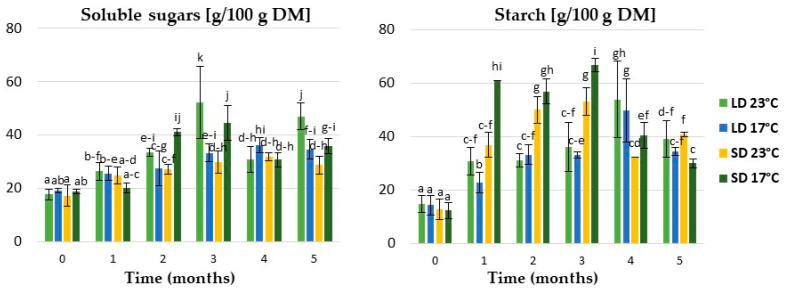
Variation in the content of soluble sugars and starch in rhubarb buds during five months of ex vitro growth under different temperatures (17 °C and 23 °C) and photoperiods (16 h and 10 h). According to Duncan’s test (*p* = 0.05), means marked with the same letter do not differ significantly (*p* = 0.05). Error bars represent standard deviation.

In all treatments, the synthesis of soluble sugars was the lowest over the first month of growth in controlled environments (Figure 4). In general, the buds’ highest content of soluble sugars was observed under LD conditions and a higher temperature (23 °C). In this treatment, the content of soluble sugars increased by 66.1% over the three months; it then decreased but increased again over the fifth month. In response to SD, underground buds showed enhanced starch accumulation by 79.7% over the first month of growth (Figure 4). The starch content remained at a high level for three months. The lower temperature (17 °C) accelerated starch accumulation and enhanced its level. Under SD conditions, enhanced starch levels coincided with growth cessation and dormancy induction. When the plantlets were grown with a 16-h photoperiod and temperature of 17 °C and 23 °C, the starch level rose only at the end of the fourth month, and then decreased. 

#### 2.1.2. Expression Analysis of Dormancy-Related Genes in Plantlets during Ex Vitro Growth under Different Photoperiods and Temperatures

The gene expression was analyzed during the five-month growth of rhubarb in the growth room under different photoperiods (16 h and 10 h) and temperatures (17 °C and 23 °C).

#### 2.1.3. Expression of Genes Related to Carbohydrate Metabolism

**Figure 5:**
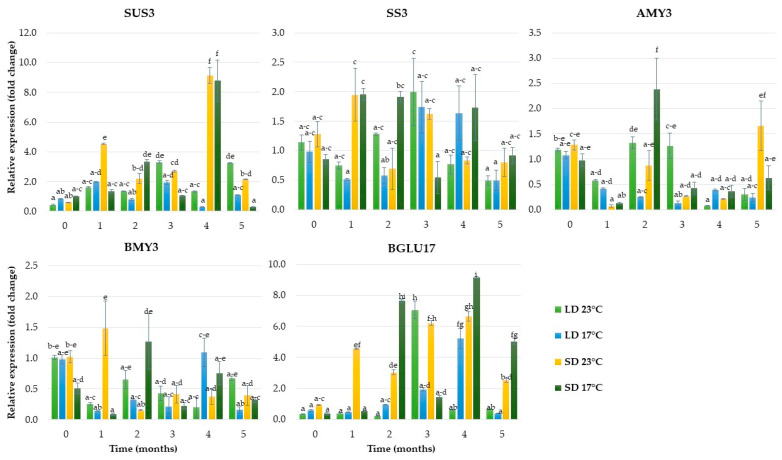
The relative expression of the genes involved in carbohydrate metabolism in rhubarb buds during five months of ex vitro growth under different temperatures (17 °C and 23 °C) and photoperiods (16 h and 10 h). According to Duncan’s test (*p* = 0.05), means marked with the same letter do not differ significantly (*p* = 0.05). Error bars represent standard deviation.

As shown in Figure 5, SD was an essential factor affecting *sucrose synthase 3* (*SUS3*) expression levels. The relative expression of this gene was upregulated, reached the maximum value after four months of growing at 23 °C and 17 °C and was almost 9.2-fold and 8.8-fold higher, respectively, than in the control. In LD conditions, the expression of the *SUS3* gene peaked over the third and fifth month of growth at 23 °C and at the end of the first month at 17 °C. The expression of the *starch synthase 3* (*SS3)* gene was enhanced rapidly over the first month of growth in SD, decreased, and then peaked again over the third month (23 °C) and fourth month (17 °C). In contrast, LD conditions resulted in low *SS3* levels at the beginning of growth ex vitro, and the highest expression levels were obtained at the end of the third month of growth at both temperatures. Among the starch amylase genes examined, *α*-*amylase* (*AMY3*) was drastically downregulated under a 16-h photoperiod and lower temperature. In turn, *β-amylase 3* (*BMY3*) was downregulated under a 16-h photoperiod and 23 °C. The gene of *β*-*glucosidase* (*BGLU17*), related to carbohydrate transport, was upregulated by both photoperiod and temperature (Figure 5).

#### 2.1.4. Expression of Genes Related to Abscisic Acid Metabolism

**Figure 6:**
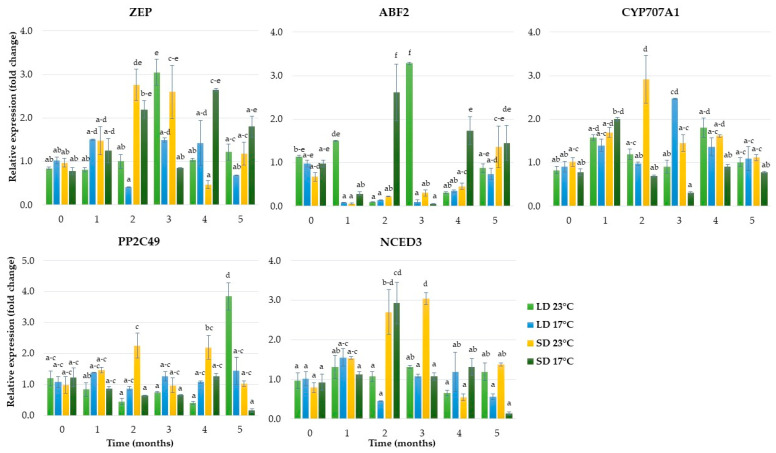
The relative expression of the genes involved in abscisic acid metabolism in rhubarb buds during ex vitro growth under different temperatures (17 °C and 23 °C) and photoperiods (16 h and 10 h). According to Duncan’s test (*p* = 0.05), means marked with the same letter do not differ significantly (*p* = 0.05). Error bars represent standard deviation.

The genes involved in ABA syntheses, such as *zeaxanthin epoxidase* (*ZEP*) and *9-cis-epoxycarotenoid dioxygenase 3* (*NCED3*), showed higher expression levels in the SD than LD conditions. In the short photoperiod, the *ZEP* was enhanced over the second month and remained high until the experiment’s end, but at different expression levels for each temperature. The *NCED3* gene was significantly upregulated over the second and third months only. In the LD condition, the expression of *ZEP* was enhanced over the third month at 23 °C, and then decreased. The gene *ABA 8′-hydroxylase* (*CYP707A1*), related to ABA catabolism, was strongly downregulated during five months of growth in SD and at a low temperature (17 °C). However, LD conditions and a low temperature increased the expression level of *CYP707A1* 2.5-fold over the third month of growth. In contrast, LD and a low temperature resulted in decreased expression levels of *ABF2*, the gene of the ABA signaling pathway. Under SD, this gene was upregulated rapidly during dormancy induction (after two months) and establishment at 17 °C. Another gene (*PP2C49*) that was involved in the ABA signaling pathway showed upregulated levels at the end of the fifth month in plantlets grown in LD and at 23 °C (Figure 6).

#### 2.1.5. Expression of Genes Related to Antioxidant Metabolism

**Figure 7:**
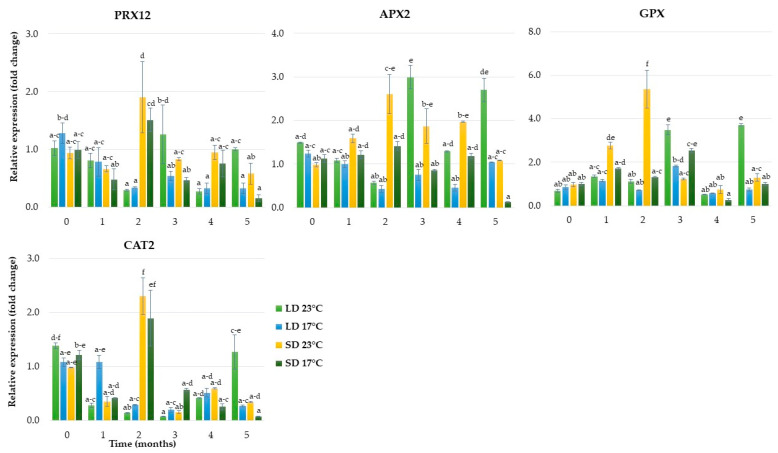
The relative expression of genes involved in antioxidant metabolism in rhubarb buds during five months of ex vitro growth under different temperatures (17 °C and 23 °C) and photoperiods (16 h and 10 h). According to Duncan’s test (*p* = 0.05), means marked with the same letter do not differ significantly (*p* = 0.05). Error bars represent standard deviation.

The genes involved in the antioxidant reaction, such as *peroxidase 12-like (PRX12), ascorbate peroxidase (APX2), glutathione peroxidase (GPX)* and *catalase 2 (CAT2)*, were upregulated rapidly in response to SD (Figure 7). The highest expression levels of these genes were observed after the second month, and they then decreased strongly over the following months. This coincided with growth cessation and dormancy induction in rhubarb plantlets. Under LD conditions, the highest activity was recorded for *GPX* and *APX2* in plantlets growing at 23 °C, but it rose after the third and fifth month and was not correlated with growth inhibition.

#### 2.1.6. Expression of Genes Related to Heat Stress

**Figure 8:**
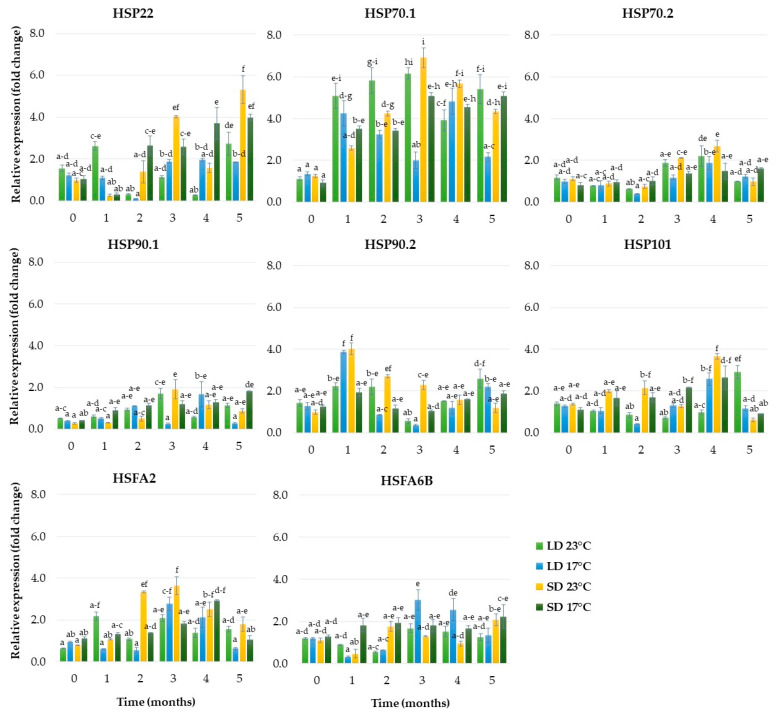
The relative expression of the genes encoding heat shock proteins and heat stress transcription factors in rhubarb buds during five months of ex vitro growth under different temperatures (17 °C and 23 °C) and photoperiods (16 h and 10 h). According to Duncan’s test (*p* = 0.05), means marked with the same letter do not differ significantly (*p* = 0.05). Error bars represent standard deviation.

As shown in Figure 8, SD was an essential factor affecting the expression levels of *HSP22* and *HSP70.1*—heat shock proteins—and *HSFA2*—heat stress transcription factor. The expression of *HSP22* was enhanced rapidly over the second month and increased until the end of the fifth month, reaching a value 5.3-fold higher than that of the control (Figure 8). The expression level of *HSFA2* significantly increased over the second month, remained at a high level until the end of the third month and then gradually decreased. The expression of the *HSP70.1* gene started to increase over the first month, was enhanced rapidly until the end of the third month, reaching a value almost 7.0-fold higher than in the control, and then slowly decreased. The *HSP70.1* gene showed also significant up-regulation in response to LD and a higher temperature (23 °C). In these conditions, the expression of the *HSP70.1* gene was enhanced rapidly over the first month and was enhanced until the end of the third month; it then decreased, but peaked again over the fifth month (Figure 8).

### 2.2. The Effect of LD and Increased Temperature (Experiment 2)

The acclimatized rhubarb plantlets were transferred to the greenhouse in early March. The effects of LD and natural temperatures in a greenhouse during a period of four months on the growth and quality of the plantlets were evaluated. The daily average/maximum temperature was 17.8/21.4 °C, 20.1/23.1 °C, 22.4/28.9 °C and 28.0/34.3 °C in March, April, May and June, respectively.

**Figure 9:**
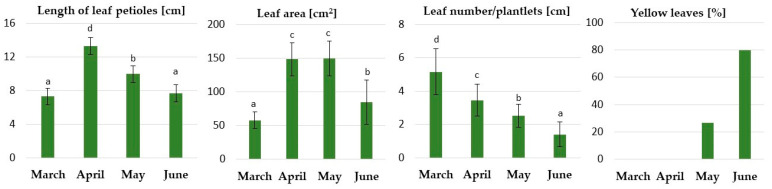
The effect of a long day and natural temperature in the greenhouse from early March to the end of June on ex vitro growth of micropropagated rhubarb ‘Raspberry’ plantlets. The daily maximum temperature was 22.3 °C, 23.1 °C, 28.9 °C and 34.0 °C in March, April, May and June, respectively. According to Duncan’s test (*p* = 0.05), the means marked with the same letter within each growth parameter do not differ significantly. Error bars represent standard deviation.

**Figure 10:**
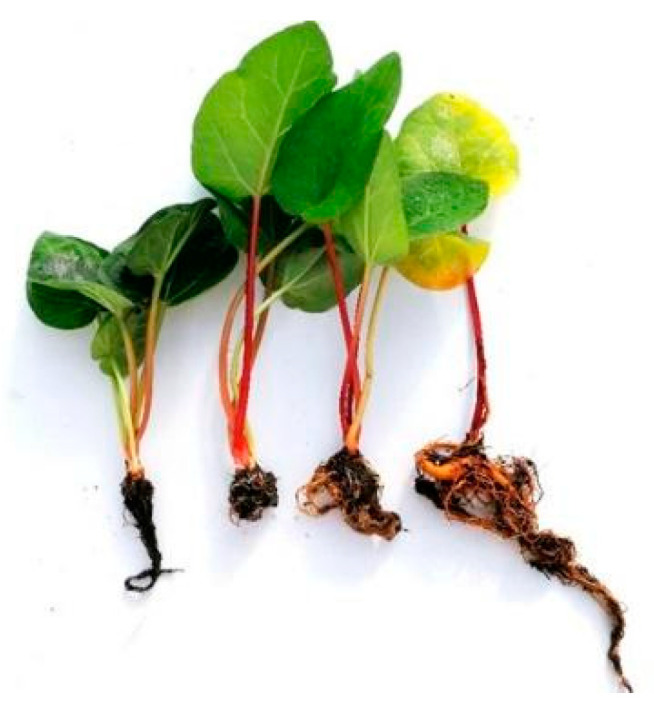
Ex vitro growth of micropropagated rhubarb plantlets after different growth periods in a greenhouse; from the left, after 1, 2, 3 and 4 months of growth in the greenhouse.

After the short adaptation period (1–2 weeks) to the greenhouse conditions, the rhubarb plantlets showed intensive growth of leaves and rhizomes (Figure 8). The highest increase in leaf petioles and blades was observed in April, at the end of the second month of growth in the greenhouse. However, the leaf number decreased. This resulted from the death of the oldest leaves developed in vitro. In May, when the maximum temperature increased to 28.9 °C (for four days), the growth rate slowed and leaf senescence was induced. A further increase in temperature in July resulted in a rapid increase in leaf senescence (Figure 9 and Figure 10) and dormancy development. At the end of June, most of the leaves (80%) were yellow.

#### 2.2.1. Physiological Responses of the Underground Buds during Dormancy Induction

To characterize the physiological changes that occurred during growth under a long day and natural temperature in the greenhouse from early March to the end of June, the content of soluble sugars, starch and abscisic acid (ABA) in the buds was determined.

**Figure 11:**
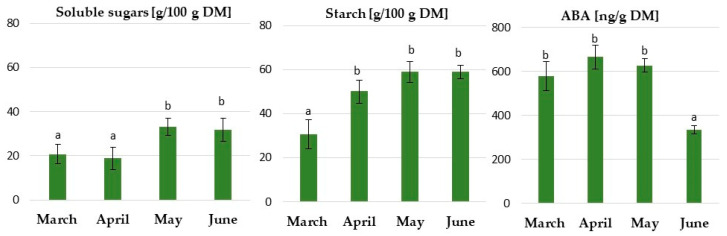
The effect of a long day and natural temperature in the greenhouse from early March to the end of June on the carbohydrate and ABA content of micropropagated rhubarb ‘Raspberry’ plantlets. Means indicated with the same letter within each carbohydrate and ABA do not differ significantly (*p* = 0.05) according to Duncan’s test. Error bars represent standard deviation.

During growth in the greenhouse, there was more starch than soluble sugars in the rhubarb buds (Figure 11). In general, the levels of carbohydrates increased over the three months of growth in the greenhouse and remained constant until the end of the experiment. At the end of May, 50% more starch than soluble sugars was found in the buds. In addition, our research found that rhubarb buds had high levels of abscisic acid already in the early stages of growth in the greenhouse. The high ABA content remained constant for three months, and then rapidly decreased over June. This coincided with dormancy establishment in the rhubarb plantlets.

#### 2.2.2. Expression Analysis of Dormancy-Related Genes in Plantlets during Ex Vitro Growth in the Greenhouse

The gene expression was analyzed after 1, 2, 3 and 4 months of growth under a long day and at a natural temperature in the greenhouse from early March to the end of June.

#### 2.2.3. Expression of Genes Related to Carbohydrate Metabolism

**Figure 12:**
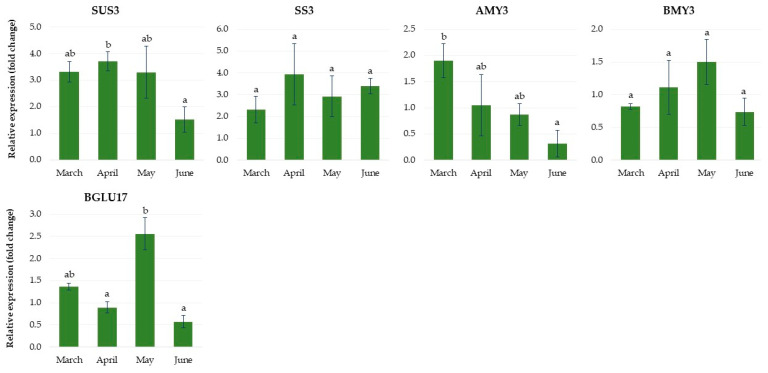
The relative expression of the genes involved in carbohydrate metabolism in rhubarb ‘Raspberry’ plantlets during ex vitro growth in the greenhouse from early March to the end of June. According to Duncan’s test (*p* = 0.05), means marked with the same letter do not differ significantly (*p* = 0.05). Error bars represent standard deviation.

As shown in Figure 12, the *sucrose synthase 3* (*SUS3*) gene was upregulated rapidly and reached the highest level at the end of April, and then its level decreased. The *starch synthase 3* (*SS3)* gene was drastically upregulated over the second month of growth in the greenhouse and remained at a high expression level until the end of June. Among the starch amylase genes examined, *α-amylase* (*AMY3*) was drastically upregulated at the end of March and then rapidly decreased. However, the expression of *β-amylase 3* (*BMY3*) was upregulated gradually and reached the highest level at the end of May. The relative expression level of the *β-glucosidase* (*BGLU17*) gene, related to carbohydrate transport and metabolism, peaked at the end of May. It then drastically decreased over the next month of growth of rhubarb plantlets in the greenhouse.

#### 2.2.4. Expression of Genes Related to Abscisic Acid Metabolism

**Figure 13:**
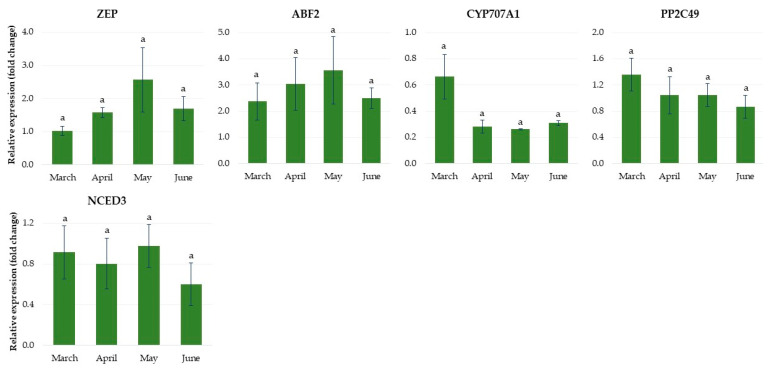
The relative expression of the genes involved in abscisic acid metabolism in rhubarb ‘Raspberry’ plantlets during ex vitro growth in the greenhouse from early March to the end of June. According to Duncan’s test (*p* = 0.05), means marked with the same letter do not differ significantly (*p* = 0.05). Error bars represent standard deviation.

The expression levels of the *ZEP* and *ABF2* genes, involved in ABA synthesis and signaling, increased over the three months of growth of rhubarb plantlets in the greenhouse, and then decreased (Figure 13). Another gene related to ABA biosynthesis, *9-cis-epoxycarotenoid dioxygenase 3* (*NCED3*), was upregulated rapidly over March, and then its expression level remained constant until the end of April, and finally it decreased again. The genes involved in ABA catabolism—*ABA 8′-hydroxylase* (*CYP707A1*)—and signaling—*phosphatase PP2C49*—showed the highest expression levels after the first month of growth in the greenhouse, and then decreased rapidly.

#### 2.2.5. Expression of Genes Related to Antioxidant Metabolism

**Figure 14:**
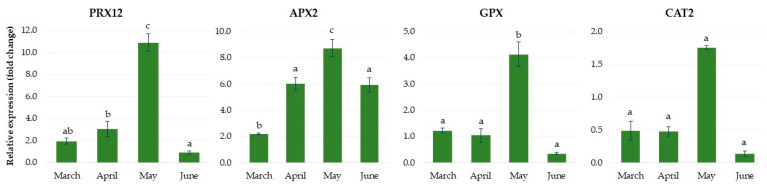
The relative expression of the genes involved in antioxidant metabolism in rhubarb ‘Raspberry’ plantlets during ex vitro growth in the greenhouse from early March to the end of June. According to Duncan’s test (*p* = 0.05), means marked with the same letter do not differ significantly (*p* = 0.05). Error bars represent standard deviation.

As shown in Figure 14, the expression levels of genes involved in the antioxidant reaction, such as *peroxidase 12-like (PRX12), glutathione peroxidase (GPX)* and *catalase 2 (CAT2)*, were low in March and April in plantlets grown in the greenhouse, but increased rapidly in May, reaching maximum values being 11.0-, 4.0- and almost 2.0-fold higher than in the controls, respectively, and they then quickly decreased in June. The *ascorbate peroxidase (APX2)* gene was upregulated rapidly in April, remained at a high expression level until the end of May and then its level also decreased.

#### 2.2.6. Expression of Genes Related to Heat Stress

**Figure 15:**
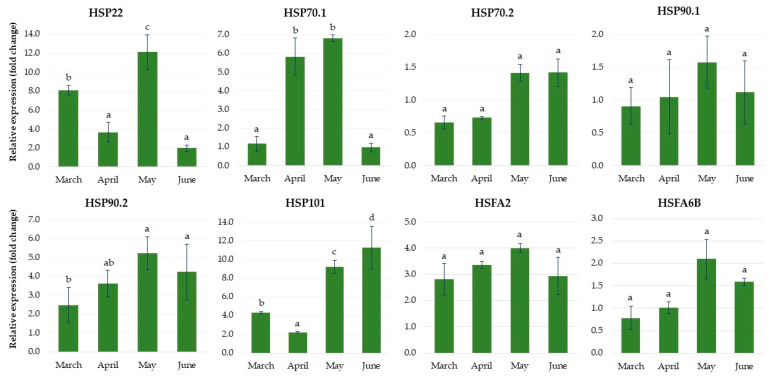
The relative expression of the genes encoding the heat shock proteins and heat stress transcription factors in rhubarb ‘Raspberry’ plantlets during ex vitro growth in the greenhouse from early March to the end of June. According to Duncan’s test (*p* = 0.05), means marked with the same letter do not differ significantly (*p* = 0.05). Error bars represent standard deviation.

As shown in Figure 15, the expression levels of genes related to heat stress, such as heat shock proteins *HSP22*, *HSP70.1* and *HSP90.2*, increased in May, reaching maximum values that were 12.0-, 7.0- and 5.0-fold higher than in controls, respectively, and they then decreased over the last month. The expression level of *HSP101* significantly increased over the four months of growth of plantlets in the greenhouse, reaching the maximum values in June, being almost 12.0-fold higher than in the control. However, the expression levels of two heat shock proteins, *HSP70.2* and *HSP90.1*, were lower than or comparable to the control. The expression levels of heat stress transcription factors, such as *HSFA2* and *HSFA6B*, increased in May, reaching maximum values 4.0- and 2.1-fold higher than in controls, respectively, and then decreased over the next month.

## 3. Discussion

The unique conditions during in vitro culture (aseptic conditions, high humidity, low irradiance, sufficient sugar and nutrients to allow heterotrophic growth) result in the formation of plantlets that differ in terms of morphology, anatomy and physiology from naturally growing plants. After ex vitro transfer, the in-vitro-derived plant material is very sensitive to various abiotic and biotic stresses [25,26]. Many herbaceous and woody perennials have developed dormancy to survive under unfavorable growth conditions [9,10,27]. Dormancy is a highly regulated and complex process and is subject to the influences of many internal and external signals. Thus far, little is known about factors that induce the dormancy of micropropagated planting material.

This study showed that micropropagated rhubarb ‘Raspberry’ plantlets are very sensitive to the photoperiod. The plantlets exposed to a 10-h photoperiod and a temperature within the normal growth range (17–23 °C) showed impaired growth of leaves and rhizomes, rapid leaf senescence and dormancy development. We did not evaluate the effect of low temperatures. Nonetheless, faster growth cessation and dormancy development at a temperature of 17 °C compared to 23 °C suggest the interaction of the photoperiod and temperature in dormancy induction in rhubarb plantlets. Similarly, in the dormancy model of plant leafy spurge, endodormancy was initiated by a decrease of the photoperiod and temperature [28]. Authors indicated that the changes occurring in controlled environments are similar to those under natural field conditions during the transition from summer to fall in the northern hemisphere [29].

In rhubarb plantlets, dormancy did not develop under LD and temperatures within the normal growth range (17–23 °C). However, the increased temperature during the greenhouse growth (in May) resulted in rapid growth cessation, leaf senescence and endodormancy development. Cooling treatment was required for the regrowth of dormant rhizomes of rhubarb ‘Raspberry’ [8]. It is known that an elevated temperature (heat stress, HS) is a significant factor limiting crop productivity and adaptation, especially when extreme temperatures coincide with the critical stage of plant growth [30,31]. Similarly to most plants from temperate climates, rhubarb has an optimum temperature of 20–25 °C for vegetative growth in the field. The yield and quality of rhubarb decrease when the average summer temperatures rise above 27–32 °C, but the plants develop endodormancy in the autumn (September—October in Poland) [8,32]. Similarly, in many fruit trees, growth cessation often occurs in summer under an increased photoperiod and temperature, whereas the establishment of endodormancy occurs in late autumn, when the days become shorter and the temperature lower. The rapid endodormancy development in micropropagated rhubarb plantlets was probably a result of their higher sensitivity to stress as compared to mature plants. Many reports showed that plants respond to adverse environmental conditions through numerous morphological, physiological, biochemical and molecular changes. However, adaptation mechanisms may differ depending on the environmental signals, developmental stage and genotype [10,33].

Changes in carbohydrate content are an important physiological marker of plant dormancy. Perennial plants exhibit strong fluctuations between dormancy stages in the rate of soluble sugars and starch production. Sugars provide cell activity energy and act as signaling molecules that regulate plant growth and development [34]. In many plant species, short days and low temperatures during autumn cause a shift in the allocation of fixed C away from soluble sugars toward starch, which acts as a reserve to support metabolism and growth [29,35,36]. Similarly, we observed a clear relationship between starch accumulation in the buds and the dormancy induction of rhubarb plantlets. The starch levels were enhanced rapidly in response to SD and high temperatures, and it was consistent with the increased expression of *starch synthase 3* (*SS3)* and downregulation of the *α-amylase* (*AMY3*) and *β-amylase 3* (*BMY3*) genes. However, after dormancy establishment in SD, the shift from starch to soluble sugars and the enhanced expression of the gene *sucrose synthase 3* (*SUS3*) were observed. These results agree with those of leafy spurge crown buds [29]. It has been demonstrated that sucrose may contribute to plant thermal adaptation by providing energy and also acting as a regulatory signal [37]. On the other hand, in the greenhouse conditions, the high starch levels in the rhubarb buds remained constant until the end of the experiment. Moreover, upregulation of *BGLU17*, involved in sugar transport, was also observed in the rhubarb buds. This suggests high metabolic activity over the endodormancy development process in rhubarb. Similar results have been reported in other plant species, including *Euphorbia esula, Paeonia lactiflora* and *Prunus mume* [36,38,39].

Plant hormones have been shown to be the most significant internal mediators in the control of dormancy development in woody and herbaceous perennials [9,10]. Among them, a crucial role in dormancy regulation is played by ABA. Moreover, it regulates many aspects of plant growth and development and is a vital messenger of stress responses [40,41]. ABA levels are increased in response to environmental signals such as low temperatures or shortened photoperiods [42]. They trigger dormancy induction in different plant species, such as *Vitis vinifera*, *Prunus persica, Pyrus pyrifolia* and *Paeonia lactiflora* [39,40,43,44]. In this study, SD and above-optimal temperatures induced the expression of ABA biosynthesis genes (*ZEP* and *NCED3*) and decreased ABA catabolism (*CYP707A1*). In the case of the plants grown in the greenhouse, the changes in ABA metabolism genes coincided with the increased levels of ABA and the transition of rhubarb plants from para- to endodormancy. In turn, genes involved in ABA signaling (*ABF3* and *PP2C49*) were upregulated in response to stress conditions (SD or increased temperature), as well as normal growth conditions (LD, optimal temperature). This indicates that ABA is essential in adapting micropropagated rhubarb plantlets to ex vitro growth. There is increasing evidence that ABA mediates the adaptation of plants to different stresses through modification of the expression status of numerous genes, including genes involved in sucrose transport and metabolism [42]. For example, ABA promotes starch accumulation in grape buds by increasing the expression of starch biosynthesis genes *SS1* and *SS3* and inhibiting genes related to starch metabolism (*INVs*) and sucrose synthesis (*SUPs*) [45]. Similarly, we observed increased ABA and starch levels in the rhubarb buds. Additionally, ABA is related to ROS generation in guard cells through the process of respiratory burst oxidase homolog (RBOH) regulation [46]. It may also improve plants’ tolerance to stress by regulating activity of the heat shock transcription factors (HSFs) and heat shock proteins (HSPs) [47]. For example, in wheat, exogenous ABA enhanced the expression of *HSP101* [48]. During the dormancy induction of rhubarb plantlets in the greenhouse, a correlation between the level of ABA and expression of HSPs and HSFs was observed. However, the detailed mechanisms await further investigation.

HFS and HSP are crucial elements of the heat shock response (HSR), a rapid response mechanism that protects plants against elevated temperatures and other stresses [49]. Their importance in the adaptation to adverse growth conditions, such as significantly elevated temperatures, was reported for different plant species, including *Sorbus* [50], tomato [51], maize [52], rice [53] and wheat [54,55,56]. Plants possess a complex regulatory network consisting of multiple *HSF* and *HSP* genes. Upon heat stress, HSPs are rapidly induced through the transcriptional activity of heat stress transcription factors (HSFs). We found that the increased temperature in the greenhouse resulted in the significant up-regulation of *HSP22*, *HSP70.1*, *HSP90.2* and *HSP101*, as well as *HSFA2* and *HSFA6B.* In addition, SD was an essential factor affecting the expression levels of the *HSP22*, *HSP70.1* and *HSFA2* genes in the rhubarb buds. However, their expression patterns varied according to the stage of dormancy development in the rhubarb buds. Crosstalk between HSFs and HSPs has been described in many papers, especially for the model plant *Arabidopsis thaliana* [31,57,58].

Under abiotic stresses, plants usually produce reactive oxygen species (ROS), which have the potential to cause oxidative damage to cells. Nevertheless, many studies have revealed ROS’s importance as a signaling molecule in mediating responses to environmental stresses [59]. Antioxidants (e.g., ascorbic acid and glutathione) and ROS-scavenging enzymes (e.g., superoxide dismutase (SOD), catalase (CAT) and glutathione peroxidase (GPX)) are essential for ROS detoxification [60]. It is believed that enhanced activity of the antioxidant system is positively correlated with high tolerance against different abiotic stresses [27,31,61]. In the present work, the genes of *peroxidase 12-like* (*PRX12*), *ascorbate peroxidase* (*APX2*) and *glutathione peroxidase* (*GPX*) showed significant upregulation in response to enhanced temperatures. In turn, SD activated *PRX12*, *APX2* and *catalase 2* (*CAT*), but their expression levels were lower than in plantlets growing in greenhouse conditions (LD and above-optimal temperature). During the ex vitro growth of micropropagated rhubarb plantlets, the enhanced expression levels of antioxidant enzymes coincided with growth cessation and dormancy induction. After dormancy establishment, genes involved in the antioxidant reaction were rapidly downregulated. Mala et al. [62] reported that in *Rheum australe*, the enhanced activity of different antioxidant enzymes in response to low temperatures may play an essential role in its adaptation to growth on the grassy or rocky slopes of the Himalayas.

To summarize, dormancy induction in micropropagated rhubarb plantlets is a stress response to unfavorable growth conditions, such as SD and high temperatures. This study showed that the dormancy of rhubarb plantlets is an adaptation strategy coordinated endogenously by the ABA, carbohydrate and ROS pathways. Knowledge of the factors affecting the ex vitro growth of micropropagated plantlets and the mechanism involved in the response to them is fundamental in the development of the successful techniques for the efficient year-round production of high-quality planting material. The results of our research can be applied to other recalcitrant herbaceous and woody plant species to improve their acclimatization and growth ex vitro.

## 4. Materials and Methods

### 4.1. Plant Material

Micropropagated planting material of a selected genotype of rhubarb ‘Raspberry’ (Polish name ‘Malinowy’) characterized by high content of anthocyanins-cyanidin-3-O-rutinoside and cyanidin-3-O-glucoside was used for the study. In vitro shoot cultures were established and multiplicated by axillary shoot growth stimulation [7]. For acclimatization, rooted shoots were transplanted to a mixture of peat bedding substrate TS1 (Klasmann-Deilmann, Warsaw, Poland) and perlite (2:1) and placed in the growth room (23 ± 2 °C; PPFD—50 µmol m^−2^ s^−1^) in plastic plug boxes covered with transparent plastic caps to prevent dehydration. The plantlets were hardened by gradually decreasing the air humidity. After six weeks of acclimatization, the rhubarb plantlets were placed in pots of 7 cm diameter and were used for experiments.

### 4.2. Ex Vitro Growth and Dormancy Induction of Plantlets

The effects of temperature and photoperiod on the growth and dormancy induction of micropropagated rhubarb plantlets were examined.

Experiment 1 was conducted in the growth room (phytotron). The rhubarb plantlets were exposed to a combination of temperatures (17 ± 1 °C and 23 ± 1 °C) and day lengths (10 h and 16 h) for five months. Light intensity of approximately 80 µmol m^−2^ s^−1^ was provided by white LED tubes (6500 K). After 1, 2, 3, 4 and 5 months, plantlets’ growth (number, length and mass of leaf petioles, and rhizome mass) was determined.

The second part of the study (Experiment 2) was carried out from early March to the end of June in a greenhouse at the National Institute of Horticultural Research, Skierniewice, Poland (WGS-84: 51.96143 N, 20.15032 E). The plantlets were grown at a natural temperature and under a 16-h photoperiod. In March and April, the natural daylight was supplemented with LED lighting at PPFD 125 µmol m^−2^ s^−1^. The daily average/maximum temperature was 17.8/21.4 °C, 20.1/23.1 °C, 22.4/28.9 °C and 28.0/34.3 °C, respectively, in March, April, May and June.

In both experiments, plantlets were manually watered as needed to maintain adequate soil moisture. Plants were fed weekly with 0.1% Kristalon (Yara, Oslo, Norway) containing 18:18:18 (*v*/*v*/*v*) NPK. After 1, 2, 3 and 4 months, plantlets’ growth (number and length of leaf petioles, leaf area) and quality (yellow leaves) were determined. Every month, ten plantlets were selected randomly and pooled to determine the content of soluble sugars, starch, abscisic acid and gene expression in the underground buds.

### 4.3. Measurements of Soluble Sugar Content

The rhubarb buds were frozen immediately after collection, and then lyophilized and homogenized. Then, bud samples (approximately 20 mg) were extracted with 1.5 mL of 80% aqueous ethanol and centrifuged at 833× *g* for 10 min. The amounts of total soluble sugars were estimated by the phenol–sulfuric method [63]. The supernatant was mixed with 5% phenol and 96% sulfuric acid. The absorbance (λ = 490 nm) of the samples was measured spectrophotometrically (Thermo Electron Corporation, Waltham, MA, USA, type Evolution 300 BB). The amounts of soluble sugars were determined against a glucose standard and expressed in grams per 100 g of dry mass (DM) plant tissue.

### 4.4. Measurements of Starch Content

Starch was determined in pellets remaining after soluble sugar analysis using a Megazyme Total Starch Assay Kit (Neogen, Lansing, MI, USA). The pellets were rinsed with ethanol, and then 3 mL of thermostable alpha-amylase solution (1/30; alpha-amylase/sodium acetate buffer, pH 5.0) was added. The samples were vortexed and placed in a boiling water bath for 12 min. The samples were allowed to cool before 100 µL of amyloglucosidase solution was added, and the samples were placed in a 50 °C water bath for 30 min. The supernatant was mixed with glucose determination reagent (GOPOD Reagent, Neogen, Lansing, MI, USA) and incubated at 50 °C for 20 min. The absorbance (λ = 510 nm) of the samples was measured spectrophotometrically (Thermo Electron Corporation Waltham, MA, USA, type Evolution 300 BB). The percentage of starch was directly calculated following the Megazyme equation based on the measured absorbance values. The analyses of the starch were performed in triplicate.

### 4.5. Quantification of Abscisic Acid

The lyophilized bud samples (approximately 25 mg) were extracted with a 1 mL mixture of methanol/water/formic acid (15/4/1; *v*/*v*/*v*) according to Dobrev and Kaminek [64], with modifications by Stefancic et al. [65]. An internal isotopic standard mixture of deuterated IAA, SA and ABA was added to each sample. The extract was then centrifuged, the supernatant was collected, and the extraction procedure was repeated. The combined supernatant was dried and reconstituted in 1 mL 1 M formic acid. This extract was fractionated with an SPE column, the Oasis MCX 1cc/30 mg (Waters, Milford, MA, USA), with 1 mL methanol, evaporated to dryness and reconstituted in 50 μL methanol. Samples prepared in this manner were analyzed on a HPLC column, the Supelco Ascentis RP-Amide (Saint Louis, MO, USA) (7.5 cm 9 4.6 mm, 2.7 μm). Mobile phases were 0.1% formic acid solution in water (solvent A) and acetonitrile/methanol (1/1) mixture. Gradient elution was applied under the flow rate of 0.5 mL/min. The HPLC apparatus was an Agilent Technologies 1260 equipped with an Agilent Technologies 6410 Triple Quad LC/MS with ESI (Electrospray Interface, Agilent Technologies, Santa Clara, CA, USA). The two most abundant secondary ions were monitored (MRM—multiple reaction monitoring modes). The primary ion for ABA (*m*/*z* 265.2) was used for quantification (quantifier ion), whereas the second (*m*/*z* 167.1) was used for additional identity confirmation (qualifier ion). Ten-point calibration curves were prepared for the analyzed compounds.

### 4.6. Molecular Analysis

Molecular studies included the expression analysis of genes related to carbohydrate, ABA and antioxidant metabolism and signal transduction pathways, as well as genes related to heat stress. According to Chang et al. [66], RNA was extracted to examine the expression of genes. DNA traces were removed from RNA samples by digestion with RQ RNase-Free DNase (Promega, Madison, WI, USA). Then, RNA samples were purified using the RNeasy Mini Kit (Qiagen, Hilden, Germany) according to the protocol for RNA clean-up. The concentration and purity of the total RNA were examined using an Epoch spectrophotometer (BioTek, Highland Park, VT, USA) in duplicate. From each sample, 1 µg of RNA was reverse-transcribed using M-MLV reverse transcriptase (Promega, Madison, WI, USA) and oligo (dT)_15_ primer (Promega, Madison, WI, USA) in a 25 µL reaction volume. Obtained cDNA samples were used for the gene expression analysis, performed using the quantitative real-time PCR (qRT-PCR) technique with specific primers. Sequences of the primers are presented in Table 1 [31,39,57,62,67,68]. Relative expression was based on the expression of the *glyceraldehyde-3-phosphate dehydrogenase* (*GAPDH*) gene, which was applied as a reference gene [62]. Quantitative RT-PCR was carried out in a Rotorgene 6000 machine (Corbett Research, Bath, United Kingdom) using the KAPA^TM^ SybrFast qPCR Master Mix (Kapa Biosystems, Amsterdam, The Netherlands), according to the manufacturer’s instructions, in a total volume of 20 µL and with 1/10 cDNA dilution for each tested sample. The annealing temperature for all primers was 58–60 °C, depending on the primer. Four ten-fold dilutions of cDNA were run with the analyzed samples to calculate the standard curve (correlation coefficient > 0.99) and the PCR efficiency. The relative quantification of the mRNA levels of tested genes was obtained from the standard curve and normalized to the reference gene and control sample. Fold change was calculated using the standard 2^−ΔΔCT^ method.

### 4.7. Statistical Analysis

The data were subjected to a three-factor (Experiment 1) and one-factor (Experiment 2) analysis of variance (ANOVA). The significance of the differences between means was evaluated by Duncan’s test at *p* = 0.05.

All the gene expression data were analyzed by using the Rotor-Gene 6000 Series Software 1.7 (Corbett Research, Bath, United Kingdom). Quantitative RT-PCR data represented the medium of at least two independent biological replications, with each performed using three technical repetitions. Standard deviation represents the variation between the biological repetitions. Microsoft Office 365 (Redmond, WA, USA) was used for figure construction.

## Figures and Tables

**Table 1:** Sequences of the primer pairs used for the real-time PCR analysis.

Gene	Sequence
*SUS3*	5′-TCGAAATTGGAGCGTCGTGA-3′
	5′-CAGTTTTCACCAAGTCGCGG-3′
*SS3*	5′-GGCTCGGCTTGTTCTAACCT-3′
	5′-TGTGTCAGTCCACATGGCTC-3′
*AMY3*	5′-CAGCGGTCTTCTTCGACCAT-3′
	5′-GCCCTGGTCCGATCTTCATT-3′
*BMY3*	5′-CAGGTACGAGGCTATCGCAG-3′
	5′-TCAGGTGATTGGTGCTCGTC-3′
*BGLU17*	5′-GAACTCAGCCACTGAGCCAT-3′
	5′-GAGTTGGACTGTAGCGGCAT-3′
*ZEP*	5′-GGCACAAGGGATCACGAACT-3′
	5′-CCTTGGAGGAGAATCGAATGG-3′
*ABF2*	5′-TCGTTGACTCTGCCTCGAAC-3′
	5′-CCTGAGCCACCTGAGACAAG-3′
*CYP707A1*	5′-CACTGAAGAGCAAGAGGCTATA-3′
	5′-TTCTTGGTATCTGCCCAACTC-3′
*PP2C49*	5′-GATCGACGACCTATCCATGCA-3′
	5′-GGTCCTCCATGGCCATCA-3′
*NCED3*	5′-TCGAAGCAGGGATGGTCAAC-3′
	5′-CCTGAGACTTTAGGCCACGG-3′
*PRX12*	5′-ATTGCTTCGTTCAGGGATGTG-3′
	5′-TCGATCGCTTCCTGTCTCAA-3′
*APX2*	5′-GGTGCCACAAGGAGCGTTCAG-3′
	5′-AAGAGCCTTGTCGGTTGGTAGTTG-3′
*GPX*	5′-CAGCCTGAGGTTCGAGCATT-3′
	5′-CACATCATTGCCACGAGCAT-3′
*CAT2*	5′-CCGGTGTTCAGACTCCTGTC-3′
	5′-AAGAGCGTGGACCATGTCAG-3′
*HSP22*	5′-TGCTATCCGATCTCTGGCTAGACC-3′
	5′-GGAGACAGAGCCACGCTTGTG-3′
*HSP70.1*	5′-TGTTGGACATTGACCTCTCTCT-3′
	5′-CGTCATCGTAGCTAAACTGGT-3′
*HSP70.2*	5′-TCATTGGTGACCCCTTTCTCT-3′
	5′-TCACATTTCTTCGAAGCTTTGTT-3′
*HSP90.1*	5′-TGGTTCTGAAAACTTCTAATATGTCG-3′
	5′-TGACACAAACCCAACCCTAGA-3′
*HSP90.2*	5′-GGACTCACCGTGCTGTCTTGTAAC-3′
	5′-ACTTGTCGTTCTTGTCTGCGTCAG-3′
*HSP101*	5′-AGGCAGGACAGTCGATTTCA-3′
	5′-CCACAATCTCGTCAAGCCTG-3′
*HSFA2*	5′-ATCATGGTGTGCTTGTAGCTGAGG-3′
	5′-AACGTCATCATCTGCTGCTGTCTC-3′
*HSFA6B*	5′-ATCGAAGAGGCGATCAGCA-3′
	5′-TGAGGATGAGGCTGCAACA-3′
*GAPDH*	5′-CTCAATGACGGCCACACAGA-3′
	5′-ACCAGTGCTGCTGGGAATG-3′

## Data Availability

All data are included in this article.
